# Mechanically Switchable Wetting Petal Effect in Self-Patterned Nanocolumnar Films on Poly(dimethylsiloxane)

**DOI:** 10.3390/nano11102566

**Published:** 2021-09-29

**Authors:** Julian Parra-Barranco, Carmen Lopez-Santos, Juan R. Sánchez-Valencia, Ana Borras, Agustin R. Gonzalez-Elipe, Angel Barranco

**Affiliations:** 1 Nanotechnology on Surfaces and Plasma Laboratory, Institute of Materials Science of Seville (CSIC-US), Américo Vespucio 49, 41092 Seville, Spain; jparrabarranco@gmail.com (J.P.-B.); jrsanchez@icmse.csic.es (J.R.S.-V.); anaisabel.borras@icmse.csic.es (A.B.); arge@icmse.csic.es (A.R.G.-E.); 2 Departamento de Física Aplicada I, Escuela Politécnica Superior, Universidad de Sevilla, Virgen de Africa, 41011 Seville, Spain; 3 Departamento de Física Atómica, Molecular y Nuclear, Facultad de Física, Universidad de Sevilla, Reina Mercedes, 41013 Seville, Spain

**Keywords:** GLAD coatings, PDMS, anisotropic wetting, droplet sliding, self-surface patterning

## Abstract

Switchable mechanically induced changes in the wetting behavior of surfaces are of paramount importance for advanced microfluidic, self-cleaning and biomedical applications. In this work we show that the well-known polydimethylsiloxane (PDMS) elastomer develops self-patterning when it is coated with nanostructured TiO_2_ films prepared by physical vapor deposition at glancing angles and subsequently subjected to a mechanical deformation. Thus, unlike the disordered wrinkled surfaces typically created by deformation of the bare elastomer, well-ordered and aligned micro-scaled grooves form on TiO_2_/PDMS after the first post-deposition bending or stretching event. These regularly patterned surfaces can be reversibly modified by mechanical deformation, thereby inducing a switchable and reversible wetting petal effect and the sliding of liquid droplets. When performed in a dynamic way, this mechanical actuation produces a unique capacity of liquid droplets (water and diiodomethane) transport and tweezing, this latter through their selective capture and release depending on their volume and chemical characteristics. Scanning electron and atomic force microscopy studies of the strained samples showed that a dual-scale roughness, a parallel alignment of patterned grooves and their reversible widening upon deformation, are critical factors controlling this singular sliding behavior and the possibility to tailor their response by the appropriate manufacturing of surface structures.

## 1. Introduction

Devices for controlling directional flows of liquids are widely utilized for self-cleaning applications [1] or, when incorporated into microfluidic devices, to control droplet transport processes [2]. Electrowetting [3] or mechanical actuation [4], the latter presenting considerable limitations in terms of reversibility and long-term reproducibility, are some of the processes utilized for such a management. To promote liquid movement, wettability must be conveniently modified, acting on both the chemistry and morphology of surfaces. Thus topographies inspired in hierarchical natural structures [5,6] and lithographic surfaces consisting of patterned lines [7,8] are commonly utilized to induce high water repellency [9] and low flow resistance [10,11,12]. Patterning using nanoparticles, [13] laser [14,15], etching [16,17] or plasma treatments [18] have been also used to tailor droplet sliding on surfaces. Similarly, a pin-release droplet effect has been reported on nanostructured thin films grown by physical vapor deposition at glancing angles (GLAD) [2]. However, those structured surfaces have a high contact angle hysteresis that precludes easy and smooth water droplets rolling off. Conversely, oil or similar liquids with low surface free energy wet well onto such surface structures, a feature complicating the surface transport of these drops [6]. This is, for example, the case of surfaces depicting the so-called petal effect found on petal surfaces of red roses and characterized by superhydrophobicity (i.e., water contact angles higher than 150°) and a high adhesive force to water [19], both originated by a particular surface micro- and nano-structure defining a Cassie impregnating wetting state.

The effect of micro and nano-patterning on the wetting and droplet movement on flexible substrates is even less understood, despite the existence of paper-based [20] as well as vermiculite-based [21] microfluidic devices for oil–water separation, or stretchable rubber fiber microchannels [22] for conductive liquids motion and tunable pillar tip nanostructures on shape memory polymers [23]. Particularly, in elastomeric surfaces of PDMS, self-generated wrinkling processes are known to induce reversible wetting, although the phenomenon is hardly controllable due to the randomness in the generation of surface structures [24] and the dependence of water adhesion on the hysteresis contact angle [25]. Nonetheless, the fabrication of mechanically switchable wetting devices relying on anisotropically structured PDMS has been attempted with different success using complex processes such as ink transfer printing [26] and 3D printing [27], laser [28,29,30], bending by magnetic induction [31], wave-like nanofibers on pre-stretched substrates [32,33], incorporation of templates [34,35], nanostructures [36,37], metallic [38,39,40] and oxide [12,41] coatings, the selective surface functionalization through plasma treatments [42] or the deposition of superhydrophobic layers [24,43,44]. In general, these procedures lack robustness and require relatively sophisticated engineering steps to ensure straightforward functionality and full reversibility upon actuation.

Previously, we have shown that linearly ordered patterned structures self-generated on PDMS substrates covered with GLAD nanocolumnar SiO_2_ thin films may act as reversible optical gratings upon mechanical actuation [45]. In the present work, we propose a modified methodology based on TiO_2_ for the fabrication of patterned elastomeric surfaces that can be reversibly modified when subjected to strain. After a first mechanical deformation of these TiO_2_/PDMS samples, the self-patterned groove structures formed at their surface conferred to them an outstanding capacity to reversibly capture and release liquid droplets by mechanical actuation. This effect can be potentially useful for droplet transport and droplet tweezing capabilities. After the careful scanning electron microscopy (SEM) and atomic force microscopy (AFM) analysis of deformed specimens, we found that a key issue for this behavior is that while the pattern layout remains inalterable upon mechanical deformation, the groove width reversibly widens in some of the strained states. The relatively easy and scalable fabrication procedure of this switchable system, its stability upon long period of time and its robustness and full reversibility upon relatively unspecific mechanical actuations, sustain its use in controlled wetting, microfluidics, wearable electronics, triboelectric nanogenerators, biomechanics and optical sensors, among other advanced applications.

## 2. Materials and Methods

### 2.1. PDMS Fabrication and TiO_2_ Thin Film Deposition by GLAD

Polidimethylsiloxane (PDMS) foils of 2 × 2 cm^2^ and 1.5 mm of thickness were fabricated by mixing Sylgard 184 (DOW) and two parts of silicon elastomer followed by degassing and curing at 80 °C for 30 min [45]. TiO_2_ thin films with 300 nm thickness were deposited on these PDMS foils in an e-beam evaporator set-up under a GLAD configuration, as previously described in [46,47] and represented in the schematic diagram of Figure S1 in the Supporting Information. A water-cooled sample holder was used to prevent radiative heating of PDMS foils during deposition of the films at a rate of 0.8–1 Ȧ/s. Samples prepared at 60° and 85° zenithal deposition angles were called 60°- and 85°-TiO_2_/PDMS, respectively. For a better understating of the reported wetting/sliding results, it is important to consider two main directions on the surface of these TiO_2_/PDMS samples: one parallel of the arrival flow of material during deposition, commonly named the *growing* direction, and perpendicular to it, usually called the *bundling* direction. This latter term refers to the association of nanocolumns observed in many GLAD thin films in a direction perpendicular to the arrival flux of material [46,47,48,49] (see Supporting Information Figure S2). This lateral partial association of the TiO_2_ nanocolumns in the TiO_2_/PDMS systems defines the direction of the grooves that appear upon mechanical deformation, as explained in the Results section.

### 2.2. Mechanical Activation and Actuation of TiO_2_/PDMS Samples

Regular grooves at the surface of TiO_2_/PDMS samples were generated when they were bent or stretched either along the *growing* or *bundling* directions, although the characteristics of the pattern (basically distance between grooves and regular distribution on the surface) were different in each case [45,49]. The TiO_2_/PDMS samples studied in the present work were mechanically activated along the *bundling* direction because the patterns were better ordered and the wetting and sliding behaviors more reliable. Once bent/stretched a first time, the samples kept memory of the generated pattern that could be reproduced so many times as the system was mechanically strained again. Characterization, wetting and drop rolling-off essays were carried out for samples in three different geometrical configurations: (1°) flat configuration after a first bending activation, (2°) concave and convex bending configurations of the samples with a curvature *κ* of 0.55 cm^−1^ and (3°) stretched configuration where a flat sample is stretched by a lateral elongation *ε* (Δ*L/L*_0_, where *L* refers to the sample length) around 0.1. The terms flat (1) concave and convex (2) and stretched (3) are kept throughout the text.

In a series of experiments, samples were dynamically stretched/bent while looking to the behavior of liquid droplets dripped and managed on their surface. In this case, the stretched sample was subjected to a larger elongation of up to 0.25.

### 2.3. Surface Characterization

Microstructure characterization was carried out with a Hitachi S-4800 Scanning Electronic Microscope (FESEM) at 2 kV. Surface topography was analyzed by Atomic Force Microscopy (AFM) using a Dulcinea microscope from Nanotec working in tapping mode and using high-frequency cantilevers. AFM images, taken on surface ranges of 40 × 40 µm^2^ and 5 × 5 µm^2^, were processed with the WSxM software freely available from Nanotec [50].

Wetting characterization was performed by dripping liquid droplets on the surface of the TiO_2_/PDMS samples in an OCA20 contact angle system from Dataphysics. Static contact angles *φ* for distilled water (WCA) and diiodomethane (DCA) were analyzed according to Young’s equation [51]. Static contact angle measurements were done with the samples in the aforementioned configurations, assuming a 3% of error that comes from the average over 3 measurements. Liquid droplets of 30 µL (water) and 5 µL (diiodomethane) were chosen for static and dynamic analysis.

Roughness, as well as structural or chemical heterogeneities, cause alterations of the ideal Young’s situation, originating a wetting contact angle hysteresis (CAH) that can be estimated through dynamic analysis, i.e., from the difference in contact angles when the volume of the deposited drop increases or decreases. These contact angles are called advancing (*φ_a_*) and receding (*φ_r_*), and the CAH, defined as the difference between *φ_a_* and *φ_r_*, is usually related to the liquid adhesion on the surface.

Surface free energy (*γ_S_*) was estimated by the Owens–Wendt–Kaelble method using the WCAs and DCAs [52] and taking into account the dispersive (*γ^d^*) and polar (*γ^p^*) components of the surface free energy according to the geometric mean method [53]:

(1) $$\gamma_{L}\left(1+cos\varphi \right)=2\left[\sqrt{\gamma_{S}^{d}\gamma_{L}^{d}}-\sqrt{\gamma_{S}^{p}\gamma_{L}^{p}}\right]$$

with *φ* the experimental contact angle and *γ_L_* the liquid surface energy. For that estimation, we used 72.8 mJ/m^2^ (*γ^p^* = 50.7 mJ/m^2^*, γ^d^* = 22.1 mJ/m^2^) and 50.8 mJ/m^2^ (*γ^p^* = 2.3 mJ/m^2^*, γ^d^* = 48.5 mJ/m^2^) as surface energy values corresponding to water and diiodomethane, respectively.

Sliding angles (*α*) were determined by tilting the sample with liquid droplets of different volume (distilled water: 2, 5, 20 and 30 μL and diiodomethane: 2 and 5 μL) deposited on its surface and looking at the angle at which they started to roll of. Measurements were taken for the three strained configurations and along two perpendicular directions: (1°) parallel to the microgrooves *α^II^*, and (2°) normal to the microgrooves *α^N^*.

## 3. Results

The study of the patterning in TiO_2_/PDMS samples and their singular sliding behavior was pursued by firstly characterizing their self-arrangement behavior upon deformation, followed with a deep analysis of their wetting behavior in the light of common chemical and roughness concepts to finally accomplish the empirical determination of wetting and sliding behaviors under static and dynamic deformations in order to discuss possible deviations from current models.

### 3.1. Self-Patterning of TiO_2_/PDMS Surfaces

**Figure 1:**
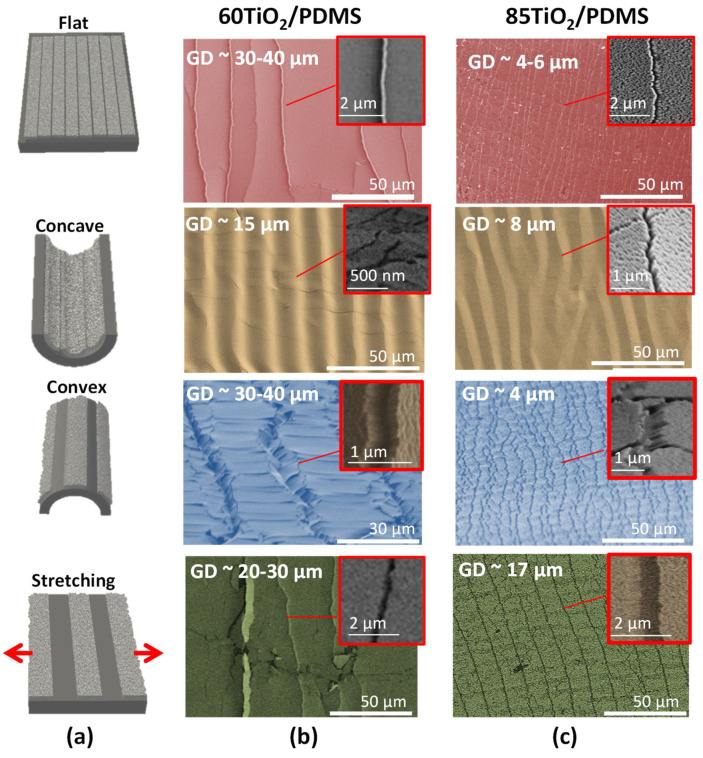
SEM analysis of the surface state of TiO_2_/PDMS samples subjected to different deformations after a first activation event by bending or stretching. (**a**) Schematic representation of the different configurations used to take the FESEM images; (**b**,**c**) FESEM micrographs of 60°-TiO_2_/PDMS (middle) and 85°-TiO_2_/PDMS surfaces (right). From top to bottom: (i) surface images of activated samples in a flat configuration after a first activation by bending; (ii,iii) concave and convex configurations during activation by bending and (iv) stretched samples in a flat configuration. The insets show magnified views of the groove regions. GD: average groove distance, as measured in the images.

The GLAD technique applied on flexible PDMS foils has demonstrated to give rise to TiO_2_ thin films with oriented nanocolumnar structures. The tilting angle of the nanocolumns in the present case was 33° and 46° for the TiO_2_/PDMS samples prepared by GLAD at deposition angles of 60° (60°-TiO_2_/PDMS sample) and 85° (85°-TiO_2_/PDMS sample), respectively (see Supporting Information Figures S1 and S2). When the “as prepared” TiO_2_/PDMS samples were firstly deformed by bending, a new structure patterned at the microscale appeared at their surfaces, as reported in Figure 1. The distance between the parallel grooves of this structure was higher for sample 60°-TiO_2_/PDMS (30–50 µm) than for sample 85°-TiO_2_/PDMS (4–6 µm) and was always independent of the applied bending curvature and film thickness. When these mechanically activated TiO_2_/PDMS samples adopted a concave configuration at a curvature of *κ*~0.55 cm^−1^, the TiO_2_ nanocolumns at the crack edges overlapped each other, leading to the generation of parallel protrusions. In turn, when the same mechanically activated samples adopted a convex configuration, microgrooves widened, forming angled valleys. It is worth stressing that surface transformation from concave to convex was completely reversible, and that the same patterned structures in Figure 1 were always obtained upon curving the samples as many times as desired. If instead of activating the “as prepared” TiO_2_/PDMS samples by bending they were firstly subjected to longitudinal stretching by an elongation *ε*~0.1, parallel grooves still formed on the surface, though more separated (i.e., 17–30 μm) than when activation was carried out by bending.

The insets in Figure 1 showing enlarged views of the generated microgrooves reveal that in the convex and stretched states, they extend up to the PDMS substrate and that the TiO_2_ nanocolumns become separated at the groove edges. They also suggest that the surface fraction of the exposed polymer substrate is higher for the convex than for the flat and concave configurations, being virtually zero in the latter. From SEM and AFM phase contrast images (reported as Figure S3 in the Supporting Information), average groove widths of around 225 nm and 340 nm could be determined for sample 85°-TiO_2_/PDMS in the convex and stretched configurations, respectively. Taking into account the average groove distances (GD), determined in Figure 1, for these two situations, it could be estimated that surface area fractions of exposed PDMS amounted to 4.5% and 6.8% for the convex and stretched configurations, respectively. Similarly, the exposed PDMS area fractions for these two configurations of sample 60°-TiO_2_/PDMS would be 2.0% and 2.3%. This means that the surface of the bent and stretched TiO_2_/PDMS samples was heterogeneous, likely affecting their wetting and sliding response when they were in these strained states. It is noteworthy in this regard that for the same sample and therefore similar pattern layout of grooves, these area fractions were much smaller in the flat un-strained states, a variation to which we attribute the switching behavior of the TiO_2_/PDMS system.

**Figure 2:**
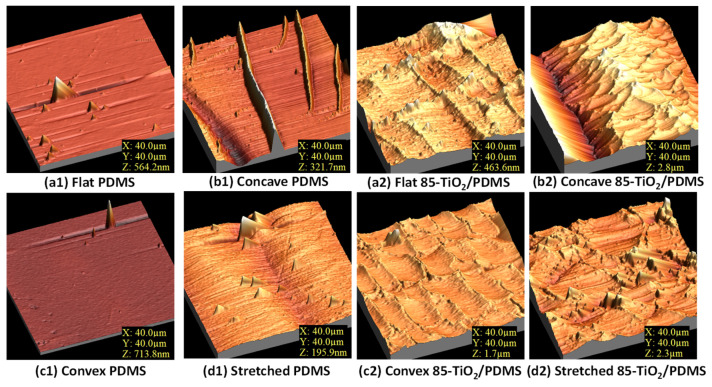
AFM images of PDMS and 85°-TiO_2_/PDMS surfaces in flat (after a single bending event, (**a1**,**a2**), stretched (**d1**,**d2**) and bent configurations, this latter in the form of either a concave (**b1**,**b2**) or a convex (**c1**,**c2**) surface, respectively.

The morphological changes evidenced by the SEM analysis were accompanied by drastic changes in surface roughness. Figure 2 shows a series of AFM images taken for the PDMS substrate and 85°-TiO_2_/PDMS sample. Clearly, the deposition of a TiO_2_ nanocolumnar layer on top of PDMS substantially contributed to generate a dual-scale roughness and increase the surface heterogeneity, particularly for the three deformed configurations (see the Z scale in the AFM images). Roughness RMS values [19] in Table 1 taken for large area images (i.e., 40 × 40 μm^2^) confirmed a roughness enhancement in the strained samples. It is worth noting that the large zone utilized for controllable wetting analysis always encompassed one of more grooves or protrusions, thus providing a realistic view of the actual surface state.

### 3.2. Wetting Behavior on Strained TiO_2_/PDMS Surfaces

Table 2 reveals that despite the significant differences in roughness and chemical composition of the surface in contact with the liquid droplet, static water contact angles (WCAs) presented little variations for the different studied samples. Larger differences were found for diiodomethane contact angles (DCAs) that substantially decreased for the three strained configurations of samples. Furthermore, surface free energies determined according to the procedure described in the experimental section depicted a considerable increase from the flat to the strained samples with significant differences depending on sample and actual configuration. According to the data in Table 2, the dispersive component, i.e., denoting Van der Waals type interactions, was the main factor responsible for this increase in surface free energy.

The dynamic contact angles determined for the studied samples yielded the contact angle hysteresis (CAH) values reported in Table 2. To a first approximation, CAHs could be used to predict adhesion forces and, indirectly, the sliding behavior of droplets. According to conventional descriptions [54], adhesion work (*W_adh_*) of liquid droplets can be defined as

(2) $$W_{adh}=\gamma_{LV}\left(\cos\varphi_{r}-\cos\varphi_{a}\right)$$

where *γ_LV_* is the liquid surface free energy, and *φ_r_* and *φ_a_* the receding and advancing contact angles, respectively. Therefore, adhesion work increases with CAHs (calculations of this parameter are reported in Table S1 in the Supporting Information). For the set of samples studied here, the obtained values did not follow a simple tendency, except for water on samples 85-TiO_2_/PDMS, where this parameter was always higher than on equivalent samples of pure PDMS. However, the relatively high CAH values in Table 2 revealed that, depending on deformation state, water droplets may adhere on these surfaces while the induced variations in this parameter upon mechanical actuation forecast the possibility to control droplet sliding.

### 3.3. Droplet Sliding on Strained TiO_2_/PDMS Surfaces

**Figure 3:**
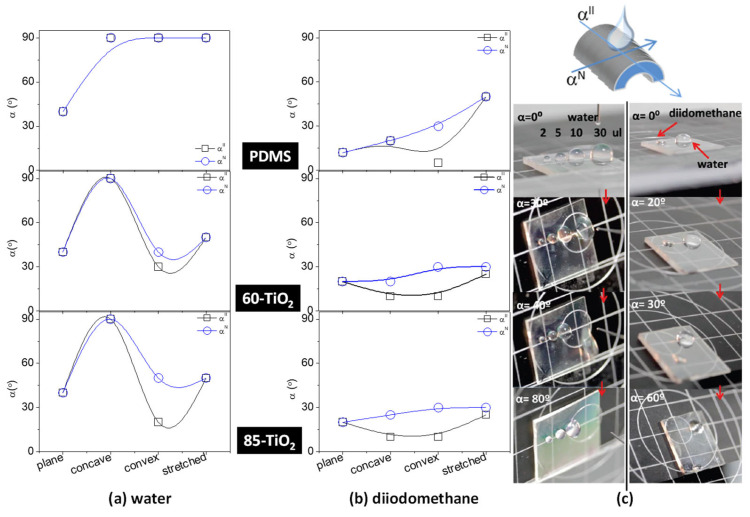
(**a**,**b**) Sliding angles (*α^II^* parallel and *α^N^* perpendicular to the direction of ordered microgrooves, respectively) vs. type of strain configuration for PDMS and 60°- and 85°-TiO_2_/PDMS samples. The volumes of the water and diiodomethane droplets were 30 µL and 5 μL, respectively. (**c**) Images showing the sliding behavior of 2, 5, 10 and 30 μL water drops left on a 60°-TiO_2_/PDMS sample at increasing tilting angles (left), and images of a 10 μL water and 5 μL diiodomethane drops left on the 85°-TiO_2_/PDMS sample at increasing tilting angles (right).

Experimentally determined rolling-off angles on TiO_2_/PDMS were quite dependent on the actual type of sample, strained configuration and, to a lesser extent, sliding direction, either normal or parallel to the grooves. Figure 3 shows a compilation of sliding angles for water and diiodomethane droplets of, respectively, 30 µL and 5 µL, determined on 60-, 85-TiO_2_/PDMS and PDMS samples subjected to strain deformation. Data for sliding directions, either parallel *α^II^* (black square) or perpendicular *α^N^* (blue circle) to the grooves, are also included. Water droplets presented a similar sliding angle around 35° on all the samples in the flat configuration, while on the strained PDMS, this parameter increased to ca. 90° independently on the deformation state. A similar increase was found for 60°- and 85°-TiO_2_/PDMS samples in the concave configuration. However, a drastic decrease was found for the stretched (ca. 40°) and, particularly, convex configurations (15° and 25° for, respectively, 85-TiO_2_ and 60-TiO_2_/PDMS samples) in the parallel sliding direction.

For the case of non-polar liquids, the sliding behavior upon mechanical actuation depicted a high sensitivity to the direction of sliding. The sliding angles of diiodomethane were smaller than 30° for the flat configuration and slightly decreased when rolling along the grooves in comparison with the direction perpendicular to them. This sliding anisotropy was comparatively improved for the bended than the stretched configurations of the TiO_2_/PDMS samples. In general, stretching the samples produces a slight enhancement of the pinning state regardless of whether the liquid is polar or non-polar. However, independently of the water droplet behavior, the surface free energy of the TiO_2_ nanostructure causes a freed movement of diiodomethane droplets if the surface is concavely deformed. This provides a selective liquid flow for this bending. The photographs in Figure 3 together with the data in Figure S4 and Video S2 in the Supporting Information also illustrate the importance of droplet weight in determining their sliding behavior; water droplets of 30 μL readily rolled-off at an angle *α* ≥ 30° on sample 60°-TiO_2_/PDMS, with a slightly favored sliding along the direction parallel to the microgrooves. It is noteworthy that despite not appreciating differences in the static contact angle along or across the groove direction, rolling-off readily occurs for water droplets of 10 µL and onwards, preferentially along such direction. This means that, for these angles and droplet volumes, gravity prevailed over adhesion interactions. Moreover, smaller diiodomethane droplets (5 μL) already slid at *α* < 30° on the 85°-TiO_2_/PDMS sample. This different sliding behavior suggests that the TiO_2_/PDMS system might be used for oil–water separation just by tilting or mechanical manipulation.

### 3.4. Dynamic Control of Droplets Sliding on Strained TiO_2_/PDMS Surfaces

**Figure 4:**
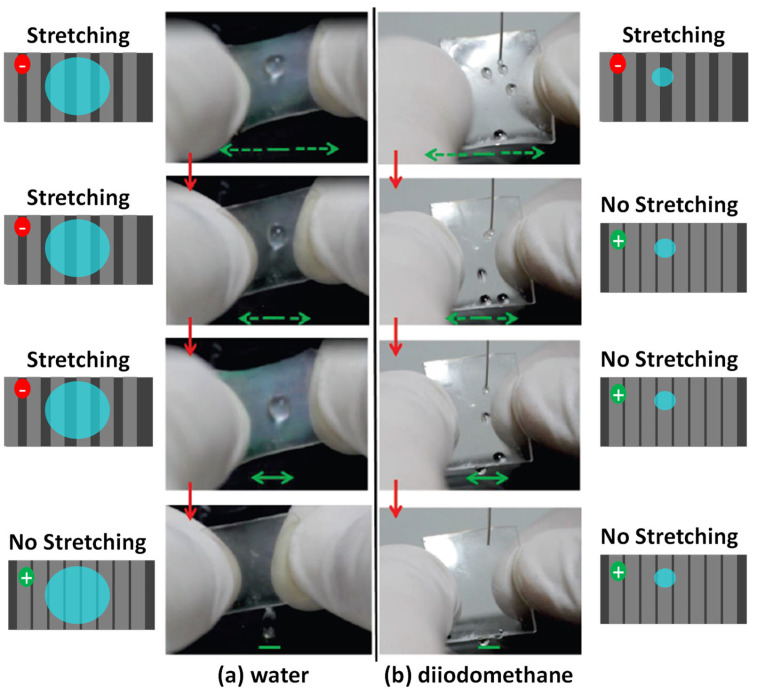
Rolling-off control of liquid droplets on TiO_2_/PDMS samples. (**a**) Photographs of 30 µL water droplet on a 85°-TiO_2_/PDMS sample in a highly stretched state (top), which is progressively released (bottom); (**b**) idem for 5 µL diiodomethane droplets on 60°-TiO_2_/PDMS sample. Green arrows indicate the direction and magnitude of stretching. Red arrows indicate the sequence of images. The schemes at the left and right sides represent the self-patterned surface for the samples in the stretched (top) and non-stretched (bottom) states, highlighting the different distribution of PDMS (dark regions in the grooves) and TiO_2_ (light regions) zones in the examined samples. Positive sliding is represented by green circles and pinned situations by red circles.

The previous experiments clearly proved a different sliding ability on mechanically deformed self-patterned TiO_2_/PDMS surfaces in a static situation. Henceforth, we show that these stretchable materials may provide the basis to develop tunable systems for a dynamic control of water droplet sliding. Figure 4 and Video S3 from Supporting Information show two examples with stretched 60°- and 85°-TiO_2_/PDMS samples and water and diiodomethane droplets of 30 μL and 5 μL, respectively. At that elongation (i.e., for *ε*~0.25, much higher than the stretching used in Figure 3), sliding of both water or diiodomethane droplets was hindered. When progressively relaxing the strain, drops started to roll off: first the diiodomethane still under a certain deformation and then water, only for the fully relaxed state. This strain dependent behavior can be qualitatively explained with the schemes in Figure 4. In the fully stretched sample, the widened cracks would produce a significant increase in the relative area of exposed PDMS in contact with the liquid and a response similar to that of this material (i.e., a sliding angle close to 90°, c.f. Figure 3). Then, when decreasing the strain and therefore the relative area of exposed PDMS, an activity identical to that in Figure 3 for unstrained samples can be obtained. Similar switchable sliding was observed when bending the samples, although experiments were less conclusive because of the practical difficulty of manually avoiding the lateral sliding of water droplets (see Supporting Information Figure S5).

Overall, the experiments reported in Figure 3 and Figure 4 clearly confirm that a controlled and switchable sliding of water and diiodomethane droplets is possible on patterned TiO_2_/PDMS surfaces. We also learned that the observed outstanding behavior was the result of the interplay between relatively complex phenomena involving the formation of self-organized ordered grooves, linked to the bundling of TiO_2_ nanocolumns in a preferential direction, the contribution of surface roughness at two length scales and the templated chemical heterogeneity of the surface.

## 4. Discussion

The experiments and results described above have been carried out with a flexible PDMS foil covered by a nanostructured TiO_2_ thin film prepared by evaporation at glancing angles. These thin films depict a nanocolumnar structure equivalent to that obtained on rigid substrates [46] and very similar to that previously reported for SiO_2_/PDMS systems prepared by a similar procedure [45]. Both in this previous work with SiO_2_ and in the present paper with TiO_2_, the nanocolumnar association character of the deposited thin film and the possibility that it offers to accommodate local stresses at the foil interface seems to be the critical factors leading to the generation of grooves upon mechanical deformation. The results herein show that the appearance of such a partially ordered groove structure is critical for the efficient and reversible control of a gradually anisotropic wetting and liquid sliding behavior on the surface of the mechanically actuated TiO_2_/PDMS system. Key features for this behavior are the appearance of a dual scale roughness of oriented nanostructures at the surface and the small average separation between patterned grooves and protrusions, the latter meaning that one or more of these features are covered by the small liquid droplets used for the experiments. Precisely, the heterogeneous character of these surfaces, exposing PDMS at the grooves and nanocolumnar TiO_2_ in the other zones, and the possibility to mechanically modulate the relative surface free energy contributions of these zones, are the main factors enabling an accurate mechanical control of the liquid–solid interaction in the strained and compressed states of the surface.

The wetting behavior of PDMS alone is known to be affected by mechanical deformation [55]. This capacity has been generally linked with rearrangement processes of surface groups affecting the partition between methyl groups and the more polar siloxane groups (Si-O), the latter being the majority in the flat sample. Rather than to this intrinsic behavior of PDMS, we attribute the singular behavior of the TiO_2_/PDMS surfaces to its mechanically controlled heterogeneous character. In this regard, the higher polar component of surface free energy found for all TiO_2_/PDMS samples with respect to PDMS can be tentatively attributed to the Ti-O (and Ti-OH) polar terminations of TiO_2_ nanocolumns.

As a direct consequence, the rolling-off of droplets appears to be mechanically controlled on the self-patterned TiO_2_/PDMS surfaces. According to classical sliding models, the rolling-off angle *α_th_* can be accounted for by the following equation:

(3) $$\sin\alpha_{\mathrm{th}}=\frac{w}{mg}\gamma_{LV}\left(\cos\varphi_{r}-\cos\varphi_{a}\right)$$

where *m* is the mass of the droplet, *g* the gravity constant, *w* the maximum width of the contact area of the liquid droplet and the surface and *γ_LV_* the liquid surface free energy. The difference between the cosine of advancing and receding contact angles is related to the adhesion work (*W_adh_*) between droplet and surface. Regarding this expression (3), the lack of clear tendencies for the CAHs (Table 2) precludes its use to predict the sliding of liquid droplets onto TiO_2_/PDMS surfaces, a feature already reported for patterned systems [56] and attributed to inherent pinning and dewetting effects on these type of surfaces [57]. Another utilized parameter to predict sliding on complex surfaces is the difference between the maximum and minimum contact angles (*φ_max_* and *φ_min_*), taken at the leading and trailing edges for a deformed drop just before sliding on an inclined surface [58]. Measurements of these parameters (see Table S2 from Supporting Information) did not predict completely the experimentally determined sliding angles for the different TiO_2_/PDMS surfaces subjected to various mechanical deformations. We assume that the main factor controlling sliding and droplet movement, overcoming the hysteresis and promoted by gravitational contributions, is a wettability driving force affected by the oriented dual-scaled surface microstructure instead of the surface free energy [26].

According to our results, while on the strained PDMS the sliding parameter increased to ca. 90° independently on the deformation state [59], on the self-patterned TiO_2_/PDMS samples, sliding depends on the strain type. Qualitatively, a simple explanation inspired by Equation (3) accounts well for the increase in rolling-off angle found for TiO_2_/PDMS samples in the concave configuration, a result that could be associated to the increase in *w* resulting from the high roughness of the developed surface in this case (Table 1) [60]. It is expected that these mechano-actuated surfaces will depict accentuated pinning effects derived from an enhanced dual-scaled roughness (Figure 1 and Figure 2), a higher density of microgrooves and the overlapping between the bundled nanocolumns of TiO_2_ resulting from this geometrical deformation (i.e., TiO_2_ bundled nanocolumns at the two sides of the groves may touch and “close” the PDMS surface under the concave configuration).

However, such an explanation would not apply to the convex configuration, where the drastic decrease in sliding angle should be discussed in the context of its surface heterogeneity and the preferential orientation of the grooves generated at the surface. Moreover, since the rolling-off angle in the direction perpendicular to the grooves was slightly higher (c.f. Figure 3) than in the opposite direction, we can conclude that the preferentially oriented microstructure of the surface contributes to guide the movement of the drops through a decrease in the sliding angle. Previous works in the literature reporting that water droplets slide better along the direction of oriented microstructures have accounted for this behavior in terms of the preservation in the continuity of the three-phase (solid–water–air) contact line (TLC) [10,61]. A comparative analysis of the sliding angles for the patterned surfaces in the convex 60°-TiO_2_/PDMS and 85°-TiO_2_/PDMS samples supports this view, contrary to what has been observed previously in rice leaf-like wavy surfaces [5]. The inter-groove distances in these samples were, respectively, 40 µm and 6 µm, meaning that roughly 88 and 550 microgrooves are in contact with the water droplets used for the analysis and that sliding should be more favorable in the latter case. It is also noteworthy that the sliding angle along the direction perpendicular to the grooves is higher for the 85°-TiO_2_/PDMS sample, in agreement with the loss of a directionality effect in this sample characterized by a high exposed ratio of PDMS (i.e., 2.0% vs. 4.5% for the 60°-TiO_2_/PDMS and 85°-TiO_2_/PDMS samples, respectively) (see Video S1 from Supporting Information). In the strained samples, the influence of differentiated TiO_2_ and PDMS zones on sliding is further confirmed by the relatively higher sliding angles found for the stretched samples (i.e., 2.3% and 6.8% of exposed PDMS for 60- and 85-TiO_2_/PDMS samples, respectively), where the lower surface density of microgrooves hampers a preeminent role of directionality for the control of droplet sliding.

These considerations support the possibility to achieve a precise control of the liquid movement through strain application, a concept that has been demonstrated previously for water droplet movement and retention [30]. In comparison with similar water adhesive–repulsive responses reported by Wong et al. [32] on switchable surfaces modified through the deposition of wave-like nanofibers on pre-stretched PDMS substrates, the TiO_2_/PDMS samples depict a better-defined pattern structure that only depends on the (highly reproducible) nanostructural characteristics of the TiO_2_ thin film prepared by GLAD and not on the stretching state of PDMS during deposition. Moreover, in agreement with the results reported by Mazaltarim et al. [62] on superhydrophobic patterned PDMS substrates, the favorable sliding found here with a marked anisotropy for the patterned nanocolumnar TiO_2_/PDMS surfaces under applied strain in a convex configuration can be described as a kind of stretching deformation modified by a negative curvature.

Diiodomethane, taken as example of a low surface tension liquid, presents an easier sliding behavior than water on the different studied surfaces. Moreover, the wetting by a non-polar liquid under strain is more directional, as evidenced by a notable anisotropic sliding that depended on whether the tension was applied in a direction parallel or perpendicular to the surface pattern. Thus, in accordance with the considerations above about the TLC, diiodomethane sliding angles slightly decreased when rolling along the groove direction in comparison with the sliding in a direction perpendicular to them. The different sliding behavior between water and low surface tension liquids suggests that the TiO_2_/PDMS system is quite appropriate as a mechanically controlled oil–water separation actuator, regulated by the interplay of surface free energy, adhesion work and gravity effects resulting from a dual scale surface roughness self-organization.

Overall, this original behavior on the TiO_2_/PDMS surfaces gives rise to a special droplet holding/releasing mechanism that could be taken as a mechanically controllable *petal* effect [19]. This behavior is different from the known transition from the *lotus* to the *petal* effects under application of a critical strain in superhydrophobic elastomeric templates [30]. The basis for the *petal*-like behavior depicted by our system resides in the management of the dual roughness reached by the combination of air-filled porous TiO_2_ nanocolumns with patterned PDMS microgrooves, giving as a result a Cassie impregnating wetting surface [62], adjustable by mechanical actuation. This mechanically tunable *petal* wettability would be useful for the transport of water droplets without the need to apply large strain deformations, as in other stretchable superhydrophobic surfaces reported previously [26,62].

## 5. Conclusions

In this work we have proved that the mechanical deformation of flexible samples consisting of oriented nanocolumnar TiO_2_ thin films deposited on PDMS produces the spontaneous formation of an ordered pattern of parallel grooves on their surface. An outstanding characteristic of these samples was that their singular patterned surface morphology, characterized by a stable groove arrangement and dimensions, can be reversibly modified by successive deformations, either bending or stretching. In addition to describing this self-organized behavior, we have demonstrated that these surface modifications can be used to reversibly control liquid wetting and droplet sliding under mechanical actuation. The fact that quite different responses were found for water and diiodomethane droplets supports the use of this type of TiO_2_/PDMS samples for separation, tweezing and transport of liquids droplets, acting as switchable systems where dynamic deformation (bending and stretching) could selectively delay and stop the motion of polar and non-polar liquids. This controllable wetting and sliding response of the micropatterned TiO_2_/PDMS surfaces is reminiscent of the typical behavior of a switchable petal effect system and sustains the fabrication of active surfaces with high prospects of use for the development of selective microfluidic devices, self-cleaning surfaces and water/oil separation purposes, among others.

## Figures and Tables

**Table 1:** RMS roughness values at two different scales determined from AFM for PDMS and patterned 60°-TiO_2_/PDMS and 85°-TiO_2_/PDMS samples in flat (after a single bending event), concave, convex and stretched configurations.

Roughness		RMS (nm) (40 μm × 40 μm)	RMS (nm) (5 μm × 5 μm)
PDMS uncoated	Flat	17	1
	Concave	39	3
	Convex	15	2
	Stretched	12	1
60°-TiO_2_/PDMS	Flat	36	12
	Concave	212	24
	Convex	110	6
	Stretched	142	8
85°-TiO_2_/PDMS	Flat	44	15
	Concave	174	26
	Convex	66	8
	Stretched	170	11

**Table 2:** Wetting behavior of PDMS and TiO_2_/PDMS surfaces: static contact angles with water (WCA) and diiodomethane (DCA), contact angles hysteresis (CAH) in brackets and surface free energy (dispersive component in brackets) of the samples under different strained configurations.

Surface Set-Up	WCA (CAH) [°]			DCA (CAH) [°]			Surface Free Energy (Dispersive c.) [mJ/m^2^]		
	PDMS	60-TiO_2_	85-TiO_2_	PDMS	60-TiO_2_	85-TiO_2_	PDMS	60-TiO_2_	85-TiO_2_
flat	112	104	100	78	84	80	23	18	19
	(64)	(53)	(73)	(30)	(17)	(31)	(19)	(14)	(15)
concave	121	121	102	60	73	63	39	29	32
	(52)	(49)	(69)	(23)	(33)	(27)	(33)	(24)	(27)
convex	112	110	112	59	57	54	37	39	41
	(29)	(33)	(63)	(30)	(44)	(35)	(32)	(33)	(35)
stretched	123	105	115	59	58	64	40	36	34
	(34)	(36)	(83)	(26)	(51)	(24)	(34)	(31)	(29)

## Data Availability

Not applicable.

## Supplementary Materials

The following are available online at www.mdpi.com/2079-4991/11/10/2566/s1, Figure S1: Schematic diagram of preparation process of the self-patterned nanocolumnar TiO_2_ surfaces on PDMS foils; Figure S2: Cross section SEM micrographs of TiO_2_ nanocolumnar films deposited at 60° (a) and 85° (b) of zenithal angle. Scheme of the growing and bundling directions according to the OAD geometry; Figure S3: Phase contrast AFM images under convex bending and stretching to identify the microgroove region of exposed PDMS on the surface of the 85-TiO_2_/PDMS sample; Figure S4: Water contact angles (WCA) and sliding angles along the parallel *(**φ^II^, α^II^*) and normal *(**φ^N^, α^N^*) directions of the microgrooves generated onto the nanocolumnar 60-TiO_2_ /PDMS sample in function of the droplet volume. Corresponding values onto a reference PDMS foil for a 30 µL water droplet are included for comparison Figure S5: Selective water adhesion on PDMS, 60°-TiO_2_/PDMS and 85°-TiO_2_/PDMS surfaces in a convex bent configuration. Pictures report the behavior on bent configuration increasing the curvature from top to bottom in each couple of panels. Drop volume was 30 μL; Table S1: Adhesion work of droplets on the different surface configurations for the TiO_2_/PDMS samples and on the PDMS substrate; Table S2: Theoretical sliding angle *α_th_* for a 30 µL water droplet from the experimental minimum (*φ_min_*) and maximum (*φ_max_*) contact angles values previous to rolling off (see image on the left). Data correspond to a flat configuration under stretching for the PDMS substrate and the 60°- and 85°-TiO_2_/PDMS surfaces. Video S1: anisotropic wetting of 60- and 85-TiO_2_/PDMS surfaces when subjected to convex bending in the parallel or perpendicular directions to the patterned microgrooves, Video S2: selective liquid droplet rolling-off on the 85-TiO_2_/PDMS surface under increasing tilting angles, Video S3: control of water and diiodomethane droplets sliding by stretching of 60- and 85-TiO_2_/PDMS surfaces on a tilted configuration.

## References

[1] Rahmawan Y., Xu L., Yang S. (2013). Self-assembly of nanostructures towards transparent, superhydrophobic surfaces. J. Mater. Chem. A.

[2] Malvadkar N.A., Hancock M.J., Sekeroglu K., Dressick W.J., Demirel M.C. (2010). An engineered anisotropic nanofilm with unidirectional wetting properties. Nat. Mater..

[3] Holmes H.R., Böhringer K.F. (2015). Transporting droplets through surface anisotropy. Microsyst. Nanoeng..

[4] Seo J., Lee S.-K., Lee J., Seung Lee J., Kwon H., Cho S.-W., Ahn J.-H., Lee T. (2015). Path-programmable water droplet manipulations on an adhesion controlled superhydrophobic surface. Sci. Rep..

[5] Lee S.G., Lim H.S., Lee D.Y., Kwak D., Cho K. (2013). Tunable Anisotropic Wettability of Rice Leaf-Like Wavy Surfaces. Adv. Funct. Mater..

[6] Kang S.M., Lee C., Kim H.N., Lee B.J., Lee J.E., Kwak M.K., Suh K.-Y. (2013). Directional Oil Sliding Surfaces with Hierarchical Anisotropic Groove Microstructures. Adv. Mater..

[7] Xu J., Hou Y., Lian Z., Yu Z., Wang Z., Yu H. (2020). Bio-Inspired Design of Bi/Tridirectionally Anisotropic Sliding Superhydrophobic Titanium Alloy Surfaces. Nanomaterials.

[8] Wu D., Wang J.-N., Wu S.-Z., Chen Q.-D., Zhao S., Zhang H., Sun H.-B., Jiang L. (2011). Three-Level Biomimetic Rice-Leaf Surfaces with Controllable Anisotropic Sliding. Adv. Funct. Mater..

[9] Watson G.S., Cribb B.W., Watson J.A. (2010). How Micro/Nanoarchitecture Facilitates Anti-Wetting: An Elegant Hierarchical Design on the Termite Wing. ACS Nano.

[10] Yoshimitsu Z., Nakajima A., Watanabe T., Hashimoto K. (2002). Effects of Surface Structure on the Hydrophobicity and Sliding Behavior of Water Droplets. Langmuir.

[11] Chung J.Y., Youngblood J.P., Stafford C.M. (2007). Anisotropic wetting on tunable micro-wrinkled surfaces. Soft Matter..

[12] Nagai H., Irie T., Takahashi J., Wakida S.-i. (2007). Flexible manipulation of microfluids using optically regulated adsorption/desorption of hydrophobic materials. Biosen. Bioelectron..

[13] Ionov L. (2012). Biomimetic 3D self-assembling biomicroconstructs by spontaneous deformation of thin polymer films. J. Mater. Chem..

[14] Hunt J.A., Williams R.L., Tavakoli S.M., Riches S.T. (1995). Laser surface modification of polymers to improve biocompatibility. J. Mater. Sci. Mater. Med..

[15] Wood M.J., Servio P., Kietzig A.-M. (2021). The Tuning of LIPSS Wettability during Laser Machining and through Post-Processing. Nanomaterials.

[16] Contraires E., Teisseire J., Sondergard E., Barthel E. (2016). Wetting against the nap—How asperity inclination determines unidirectional spreading. Soft Matter..

[17] Formentin P., Marsal L.F. (2021). Hydrophobic/Oleophilic Structures Based on MacroPorous Silicon: Effect of Topography and Fluoroalkyl Silane Functionalization on Wettability. Nanomaterials.

[18] Houston K.S., Weinkauf D.H., Stewart F.F. (2002). Gas transport characteristics of plasma treated poly (dimethylsiloxane) and polyphosphazene membrane materials. J. Membr. Sci..

[19] Feng L., Zhang Y., Xi J., Zhu Y., Wang N., Xia F., Jiang L. (2008). Petal effect: A superhydrophobic state with high adhesive force. Langmuir.

[20] Li C., Boban M., Snyder S.A., Kobaku S.P.R., Kwon G., Mehta G., Tuteja A. (2016). Paper-Based Surfaces with Extreme Wettabilities for Novel, Open-Channel Microfluidic Devices. Adv. Funct. Mater..

[21] Cuoung D., Bui T.T., Cho Y.B., Kim Y.S. (2021). Highly Hydrophobic Polydimethylsiloxane-Coated Expanded Vermiculite Sorbents for Selective Oil Removal from Water. Nanomaterials.

[22] Guan L., Nilghaz A., Su B., Jiang L., Cheng W., Shen W. (2016). Stretchable-Fiber-Confined Wetting Conductive Liquids as Wearable Human Health Monitors. Adv. Funct. Mater..

[23] Lai H., Shang Y., Cheng Z., Lv T., Zhang E., Zhang D., Wang J., Liu Y. (2019). Control of tip nanostructure on superhydrophobic shape memory arrays toward reversibly adjusting water adhesion. Adv. Compos. Hybrid. Mater..

[24] Lin P.-C., Yang S. (2009). Mechanically switchable wetting on wrinkled elastomers with dual-scale roughness. Soft Matter..

[25] Wang C., Nair S.S., Veeravalli S., Moseh P., Wynne K.J. (2016). Sticky or Slippery Wetting: Network Formation Conditions Can Provide a One-Way Street for Water Flow on Platinum-cured Silicone. ACS Appl. Mater. Interfaces.

[26] Park J.K., Yang Z., Kim S. (2017). Black Silicon/Elastomer Composite Surface with Switchable Wettability and Adhesion between Lotus and Rose Petal Effects by Mechanical Strain. ACS Appl. Mater. Interfaces.

[27] Wang B., Zhang Z., Pei Z., Qiu J., Wang S. (2020). Current progress on the 3D printing of thermosets. Adv. Compos. Hybrid. Mater..

[28] Shin J., Ko J., Jeong S., Won P., Lee Y., Kim J., Hong S., Jeon N.L., Ko S.H. (2021). Monolithic digital patterning of polydimethylsiloxane with successive laser pyrolysis. Nat. Mater..

[29] Qi L., Ruck C., Spychalski G., King B., Wu B., Zhao Y. (2018). Writing Wrinkles on Poly(dimethylsiloxane) (PDMS) by Surface Oxidation with a CO2 Laser Engraver. ACS Appl Mater. Interfaces.

[30] Wang J.-N., Liu Y.-Q., Zhang Y.-L., Feng J., Wang H., Yu Y.-H., Sun H.-B. (2018). Wearable Superhydrophobic Elastomer Skin with Switchable Wettability. Adv. Funct Mater..

[31] Wang H., Zhang Z., Wang Z., Liang Y., Cui Z., Zhao J., Li X., Ren L. (2019). Multistimuli-Responsive Microstructured Superamphiphobic Surfaces with Large-Range, Reversible Switchable Wettability for Oil. ACS Appl. Mater. Interfaces.

[32] Wong W.S.Y., Gutruf P., Sriram S., Bhaskaran M., Wang Z., Tricoli A. (2016). Strain Engineering of Wave-like Nanofibers for Dynamically Switchable Adhesive/Repulsive Surfaces. Adv. Funct. Mater..

[33] Lu C., Li H., Yu S., Jiao Z., Li L. (2020). Ridged Zn/PDMS smart surface with wide-range reversible wettability and high sensitivity responsive to mechanical strain. Mater. Des..

[34] Han X., Hou J., Xie J., Yin J., Tong Y., Lu C., Möhwald H. (2016). Synergism of Dewetting and Self-Wrinkling To Create Two-Dimensional Ordered Arrays of Functional Microspheres. ACS Appl. Mater. Interfaces.

[35] Mishra H., Schrader A.M., Lee D.W., Gallo A., Chen S.-Y., Kaufman Y., Das S., Israelachvili J.N. (2016). Time-Dependent Wetting Behavior of PDMS Surfaces with Bioinspired, Hierarchical Structures. ACS Appl. Mater. Interfaces.

[36] Zhou S., Ding X., Wu L. (2013). Fabrication of ambient-curable superhydrophobic fluoropolysiloxane/TiO2 nanocomposite coatings with good mechanical properties and durability. Prog. Org. Coat..

[37] Wang Y., Lai H., Cheng Z., Zhang H., Liu Y., Jiang L. (2019). Smart Superhydrophobic Shape Memory Adhesive Surface toward Selective Capture/Release of Microdroplets. ACS Appl. Mater. Interfaces.

[38] Hoshian S., Jokinen V., Franssila S. (2016). Robust hybrid elastomer/metal-oxide superhydrophobic surfaces. Soft Matter..

[39] Qi D., Zhang K., Tian G., Jiang B., Huan Y. (2021). Stretchable Electronics Based on PDMS Substrates. Adv. Mater..

[40] Yu S., Ma L., Sun Y., Lu C., Zhou H., Ni Y. (2019). Controlled Wrinkling Patterns in Periodic Thickness-Gradient Films on Polydimethylsiloxane Substrates. Langmuir.

[41] Nakata K., Udagawa K., Ochiai T., Sakai H., Murakami T., Abe M., Fujishima A. (2011). Rapid erasing of wettability patterns based on TiO2-PDMS composite films. Mater. Chem. Phys..

[42] Li Z., Liu Y., Marin M., Yin Y. (2020). Thickness-dependent wrinkling of PDMS films for programmable mechanochromic responses. Nano Res..

[43] Lee E., Zhang M., Cho Y., Cui Y., Van der Spiegel J., Engheta N., Yang S. (2014). Tilted pillars on wrinkled elastomers as a reversibly tunable optical window. Adv. Mater..

[44] Kim K.-D., Seo H.O., Sim C.W., Jeong M.-G., Kim Y.D., Lim D.C. (2013). Preparation of highly stable superhydrophobic TiO2 surfaces with completely suppressed photocatalytic activity. Prog. Org. Coat..

[45] Parra-Barranco J., Oliva-Ramirez M., Gonzalez-Garcia L., Alcaire M., Macias-Montero M., Borras A., Frutos F., Gonzalez-Elipe A.R., Barranco A. (2014). Bending induced self-organized switchable gratings on polymeric substrates. ACS Appl. Mater. Interfaces.

[46] Gonzalez-García L., Parra-Barranco J., Sanchez-Valencia J.R., Ferrer J., Garcia-Gutierrez M.-C., Barranco A., Gonzalez-Elipe A.R. (2013). Tuning dichroic plasmon resonance modes of gold nanoparticles in optical thin films. Adv. Funct. Mater..

[47] González-García L., Barranco A., Páez A.M., González-Elipe A.R., García-Gutiérrez M.-C., Hernández J.J., Rueda D.R., Ezquerra T.A., Babonneau D. (2010). Structure of glancing incidence deposited TiO2 thin films as revealed by grazing incidence small-angle x-ray scattering. ChemPhysChem.

[48] Sanchez-Valencia J.R., Toudert J., Borras A., Barranco A., Lahoz R., De La Fuente G.F., Frutos F. (2011). Gonzalez-Elipe, A.R. Selective dichroic patterning by nanosecond laser treatment of ag nanostripes. Adv. Mater..

[49] Barranco A., Borras A., Gonzalez-Elipe A.R., Palmero A. (2016). Perspectives on oblique angle deposition of thin films: From fundamentals to devices. Prog. Mater. Sci..

[50] Horcas I., Fernández R., Gómez-Rodríguez J.M., Colchero J., Gómez-Herrero J., Baro A.M. (2007). WSXM: A software for scanning probe microscopy and a tool for nanotechnology. Rev. Sci. Instrum..

[51] Young T. (1805). An Essay on the Cohesion of Fluids. Philos. Trans. R. Soc. Lond.

[52] Owens D.K., Wendt R.C. (1969). Estimation of the surface free energy of polymers. J. Appl. Polym. Sci..

[53] Ma K.-X., Chung T.-S. (2001). Effect of −C(CF3)2− on the Surface Energy of Main-Chain Liquid Crystalline and Crystalline Polymers. J. Phys. Chem. B.

[54] Roucoules V., Ponche A., Geissler A., Siffer F., Vidal L., Ollivier S., Vallat M.F., Marie P., Voegel J.C., Schaaf P. (2007). Changes in silicon elastomeric surface properties under stretching induced by three surface treatments. Langmuir.

[55] Good R.J. (1992). Contact angle, wetting, and adhesion: A critical review. J. Adhes Sci. Technol..

[56] Krasovitski B., Marmur A. (2005). Drops down the hill: Theoretical study of limiting contact angles and the hysteresis range on a tilted plate. Langmuir.

[57] Das P.K., Grippin A., Kwong A., Weber A.Z. (2012). Liquid-Water-Droplet Adhesion-Force Measurements on Fresh and Aged Fuel-Cell Gas-Diffusion Layers. J. Electrochemical. Soc..

[58] Pierce E., Carmona F.J., Amirfazli A. (2008). Understanding of sliding and contact angle results in tilted plate experiments. Colloids Surf. A-Physicochem Eng. Asp..

[59] Huang J., Cai Y., Xue C., Ge J., Zhao H., Yu S.-H. (2021). Highly stretchable, soft and sticky PDMS elastomer by solvothermal polymerization process. Nano Res..

[60] Kanungo M., Mettu S., Law K.-Y., Daniel S. (2014). Effect of roughness geometry on wetting and dewetting of rough PDMS surfaces. Langmuir.

[61] Yong J., Yang Q., Chen F., Zhang D., Farooq U., Du G., Hou X. (2014). A simple way to achieve superhydrophobicity, controllable water adhesion, anisotropic sliding, and anisotropic wetting based on femtosecond-laser-induced line-patterned surfaces. J. Mater. Chem. A.

[62] Roy P.K., Ujjain S.K., Dattatreya S., Kumar S., Pant R., Khare K. (2019). Mechanically tunable single-component soft polydimethylsiloxane (PDMS)-based robust and sticky superhydrophobic surfaces. Appl. Phys. A.

