# Supplementary material for: Mechanically Switchable Wetting Petal Effect in Self-Patterned Nanocolumnar Films on Poly(dimethylsiloxane)
Source: Nanomaterials (Basel). 2021 Sep 29;11(10):2566. doi: 10.3390/nano11102566 (PMC8538580; doi:10.3390/nano11102566)
Supplement: Supplementary file 1 [file nanomaterials-11-02566-s001.zip › Supplementary Materials.pdf]

Supporting Information

Mechanically Switchable Wetting Petal Effect in Self-Patterned Nanocolumnar Films on Poly(dimethylsiloxane)

Julian Parra-Barranco [1], Carmen Lopez-Santos [1, 2, *], Juan R. Sánchez-Valencia[1, 3], Ana Borras [1], Agustin R. Gonzalez-Elipe [1] and Angel Barranco [1,*]

Figure S1. Schematic diagram of preparation process of the self-patterned nanocolumnar TiO_2 surfaces on PDMS foils

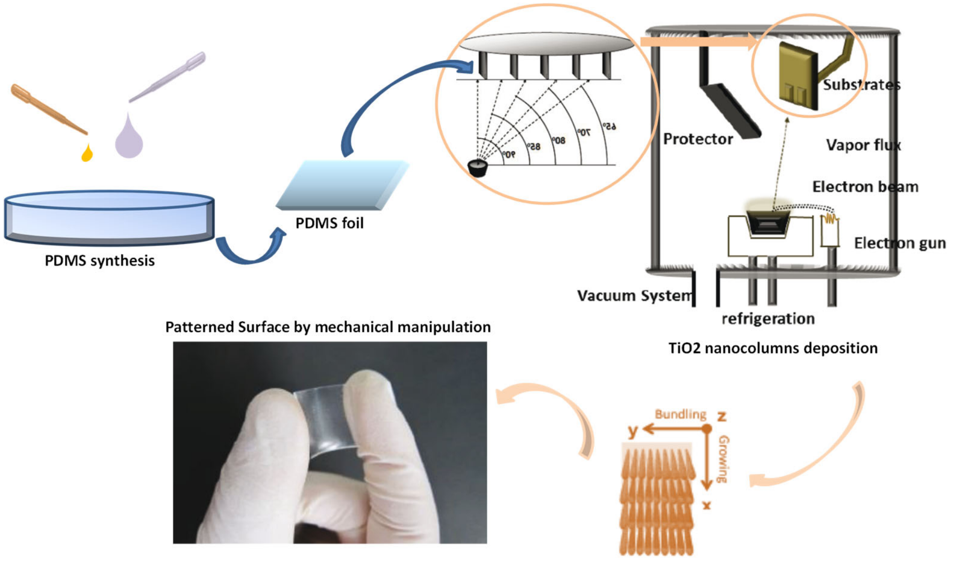

Figure S2. Cross section SEM micrographs of TiO_2 nanocolumnar films deposited at 60º (a) and 85º (b) of zenithal angle. Scheme of the growing and bundling directions according to the GLAD geometry.

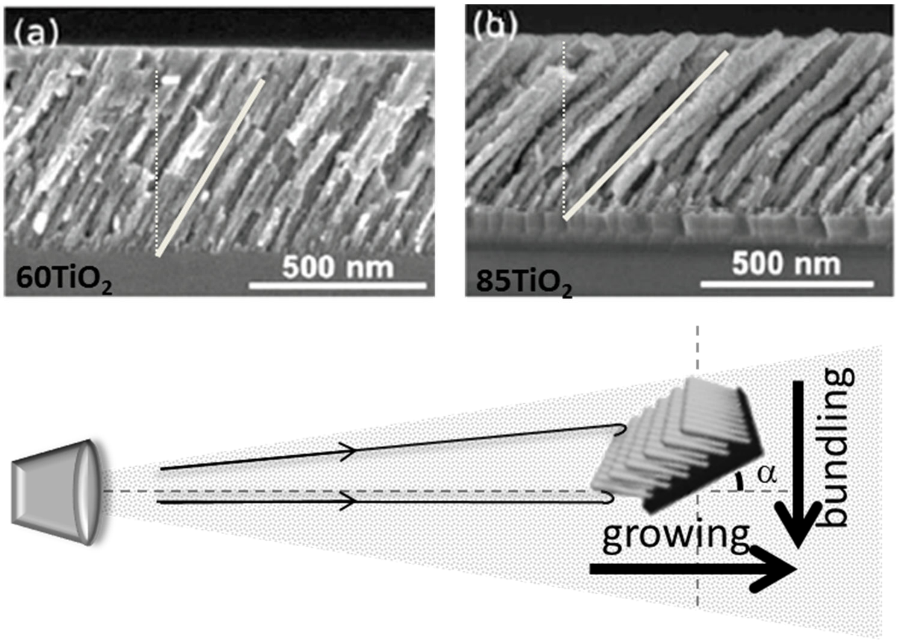

Figure S3. Phase contrast AFM images under convex bending and stretching to identify the microgroove region of exposed PDMS on the surface of the 85-TiO_2/PDMS sample.

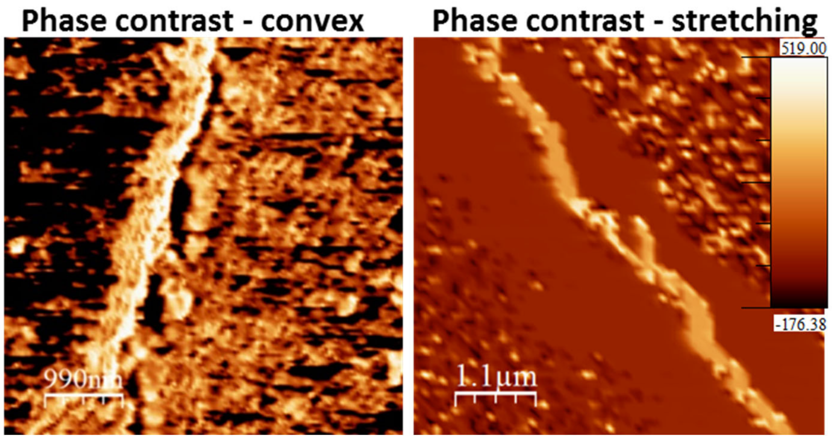

Figure S4. Water contact angles (WCA) and sliding angles along the parallel (φ^{II}, α^{II}) and normal (φ^{N}, α^{N}) directions of the microgrooves generated onto the nanocolumnar 60-TiO_2 /PDMS sample in function of the droplet volume. Corresponding values onto a reference PDMS foil for a 30µl water droplet are included for comparison.

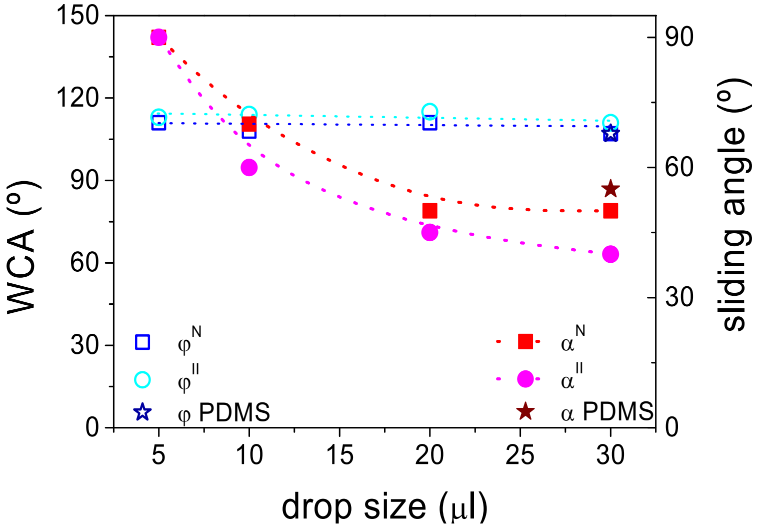

Figure S5. Selective water adhesion on PDMS, 60º-TiO_2/PDMS and 85º-TiO_2/PDMS surfaces in a convex bent configuration. Pictures report the behavior on bent configuration increasing the curvature from top to bottom in each couple of panels. Drop volume was 30 μl.

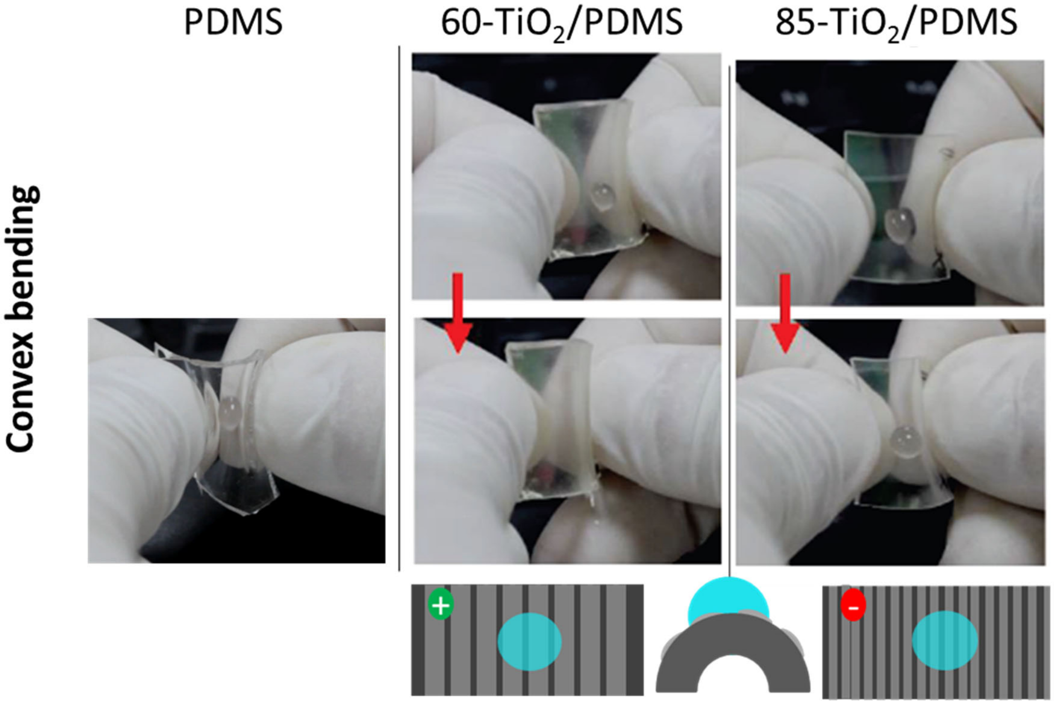

Table S1. Adhesion work of droplets on the different surface configurations for the TiO_2/PDMS samples and on the PDMS substrate.

Surface set-up	Water adhesion work [mN/m]			Diiodomethane adhesion work [mN/m]		
	PDMS	60-TiO_2	85-TiO_2	PDMS	60-TiO_2	85-TiO_2
**flat**	76	63	77	23	15	25
**concave**	64	60	82	15	24	18
**convex**	36	41	75	18	22	18
**stretched**	41	45	92	17	24	17

Table S2. Theoretical sliding angle α_{th} for a 30µl water droplet from the experimental minimum (φ_{min}) and maximum (φ_{max}) contact angles values before rolling off (see image on the left). Data correspond to a flat configuration under stretching for the PDMS substrate and the 60º- and 85º- TiO_2/PDMS surfaces.

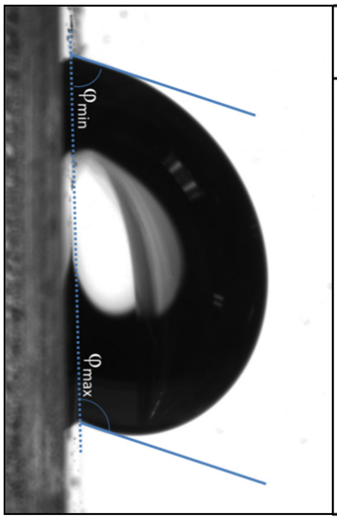

sample		φ_{min} (º)	φ_{max} (º)	α_{th} (º)
PDMS	flat	79	118	36
	stretched	94	123	27
60-TiO_2	flat	83	126	41
	stretched	74	96	21
85-TiO_2	flat	90	121	29
	stretched	60	87	23
